# Supplementary material for: The Leukotriene Receptor Antagonist Montelukast Attenuates Neuroinflammation and Affects Cognition in Transgenic 5xFAD Mice
Source: Int J Mol Sci. 2021 Mar 9;22(5):2782. doi: 10.3390/ijms22052782 (PMC7967180; doi:10.3390/ijms22052782)
Supplement: Supplementary file 1 [file ijms-22-02782-s001.zip › supplementary/ijms-1097146 supl.pdf]

The leukotriene receptor antagonist Montelukast ameliorates neuroinflammation and improves cognition in a mouse model of Alzheimer's disease

Johanna Michael [1,2], Julia Zirknitzer [1,2], Michael Unger [1,2], Rodolphe Poupardin [2], Tanja Rieß [1,2], Nadine Paiement [3], Horst Zerbe [3], Birgit Hutter-Paier [4], Herbert Reitsamer [6], Ludwig Aigner [1,2,5]

Supplementary figures

Gene expression analysis sorted by Sex

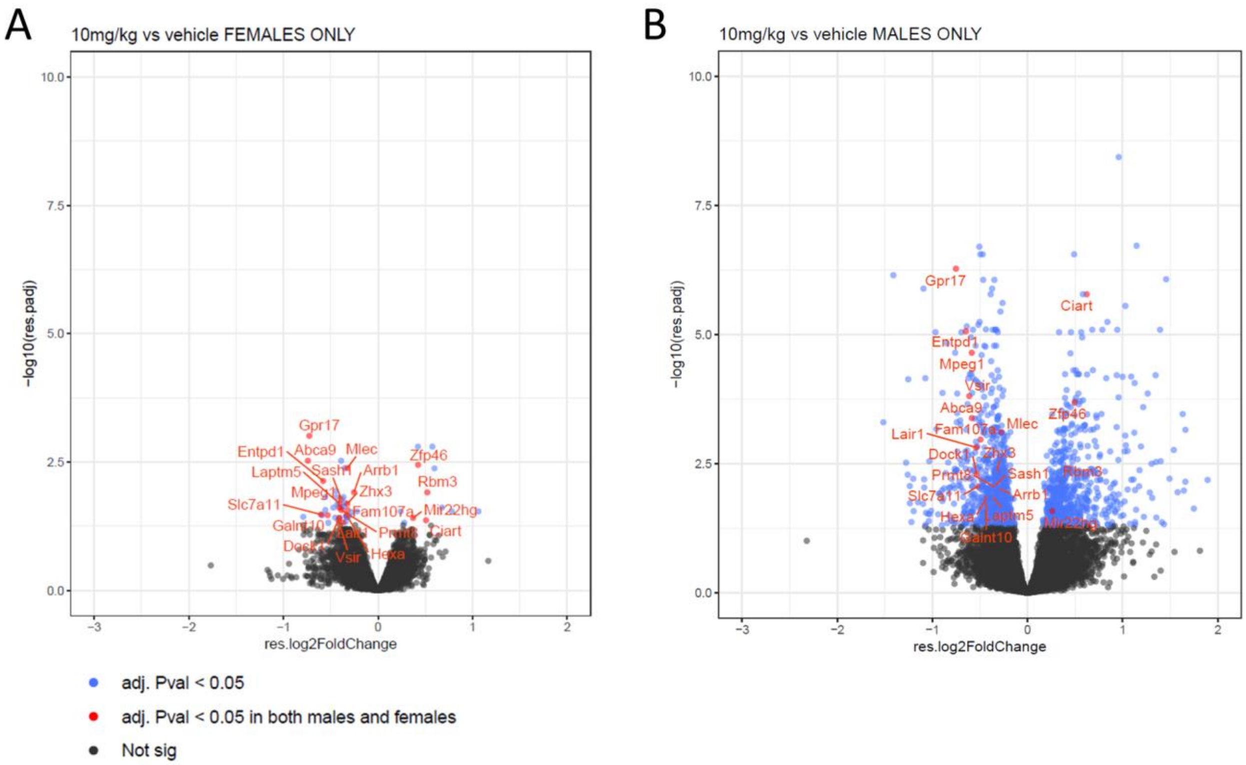

Supplementary figure 1 Gene expression data from hippocampal tissue of high-dose MTK vs vehicle treated mice, separated by sex. (a) Volcano plot showing significantly DEGs in female mice. (b) Volcano plot showing significantly DEGs in male mice. Red genes are significantly differentially expressed in both datasets.

Histological analysis of cortical microglia

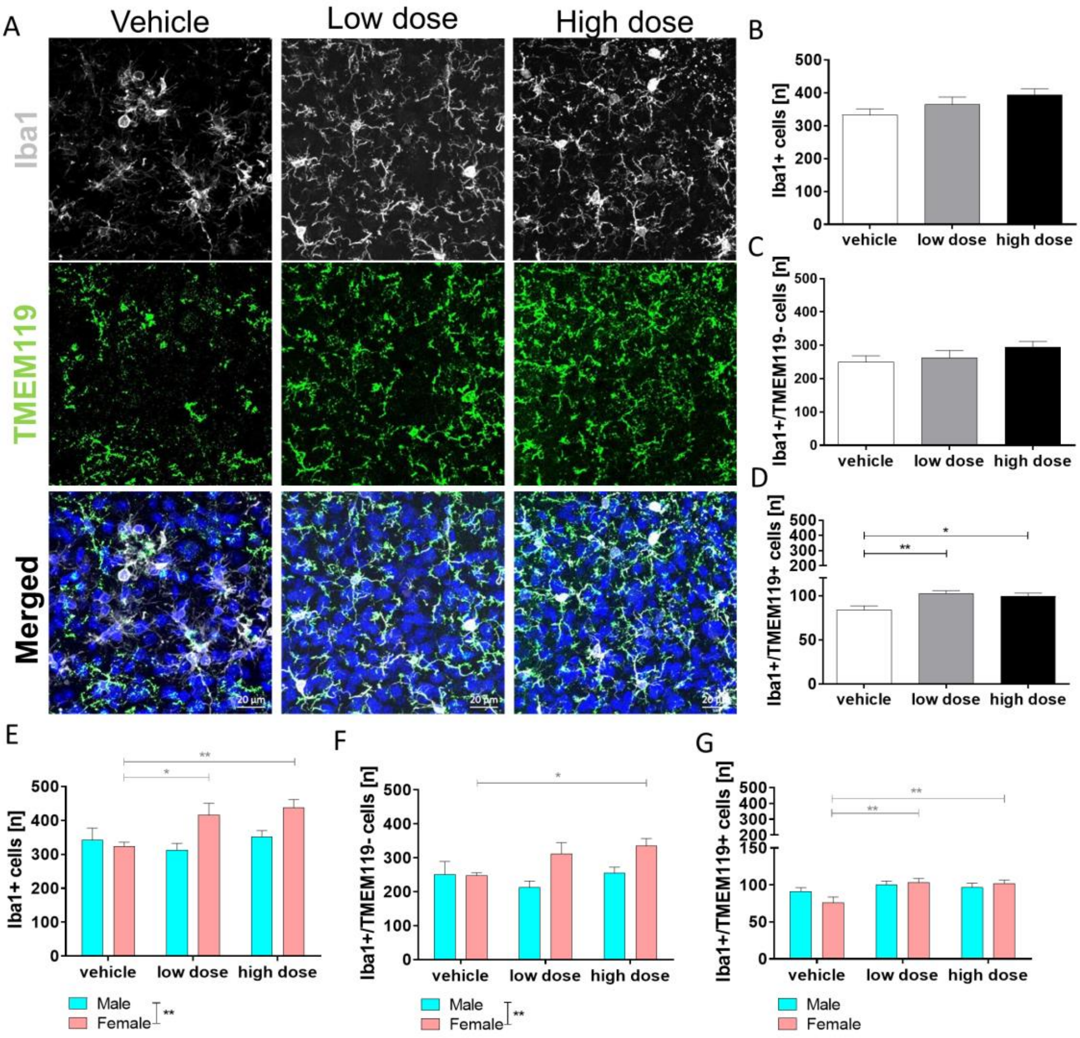

Supplementary figure 2 Immunohistochemical analysis of microglia with and without MTK treatment in the cortex. (a) Representative images of microglia positive for Iba1 and Tmem119.; scalebar:20µm (b) We observed no significant difference in numbers of Iba1+ microglia with MTK. (c) No significant difference in numbers of Iba1+/Tmem119- cells between groups. (d) We observed a significant increase in numbers of Iba1+/Tmem119+ microglia with MTK. (e) The increase in Iba1+ cells comes from female mice, which have significantly more microglia than male mice of the same age. (f) Numbers of cells separated by sex. Female mice have significantly more Iba1+/Tmem119- cells than male mice. (g) The increase in subpopulation comes from females, which have less of these cells in the vehicle group but increase to more of these cells in both MTK treatment groups. Data are shown as mean +/- SEM. One-way ANOVA was performed with Tukey´s post hoc test. P-values < 0.05 were considered significant.

Activation state analysis of cortical microglia

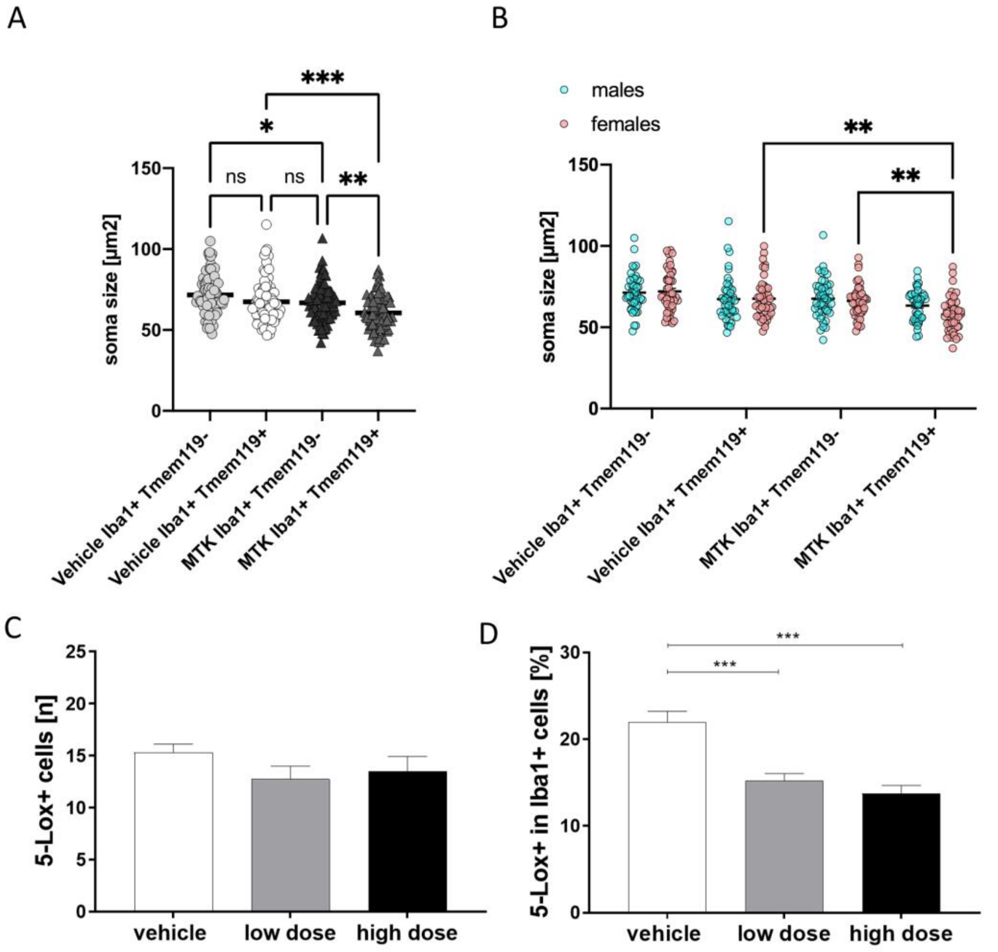

Supplementary figure 3 (a) MTK reduces soma-sizes in the Iba1+/Tmem119- and in the Iba1+/Tmem119+ subpopulation in the cortex. (b) Sex-specific soma-size analysis: Effects of MTK on soma-size are seen in females, but also in Iba1+/Tmem119+ cells in males. (c) Quantification of 5-Lox positive cells. There was no significant difference between groups (d) percentage of 5-Lox+ cells in the Iba1+ microglia population, showing a significant reduction in percentage of 5-Lox+ microglia. There was no significant difference between male and female mice in this analysis. Data are shown as mean +/- SEM. One-way ANOVA was performed with Tukey´s post hoc test. P-values < 0.05 were considered significant.

Histological analysis of plaque pathology

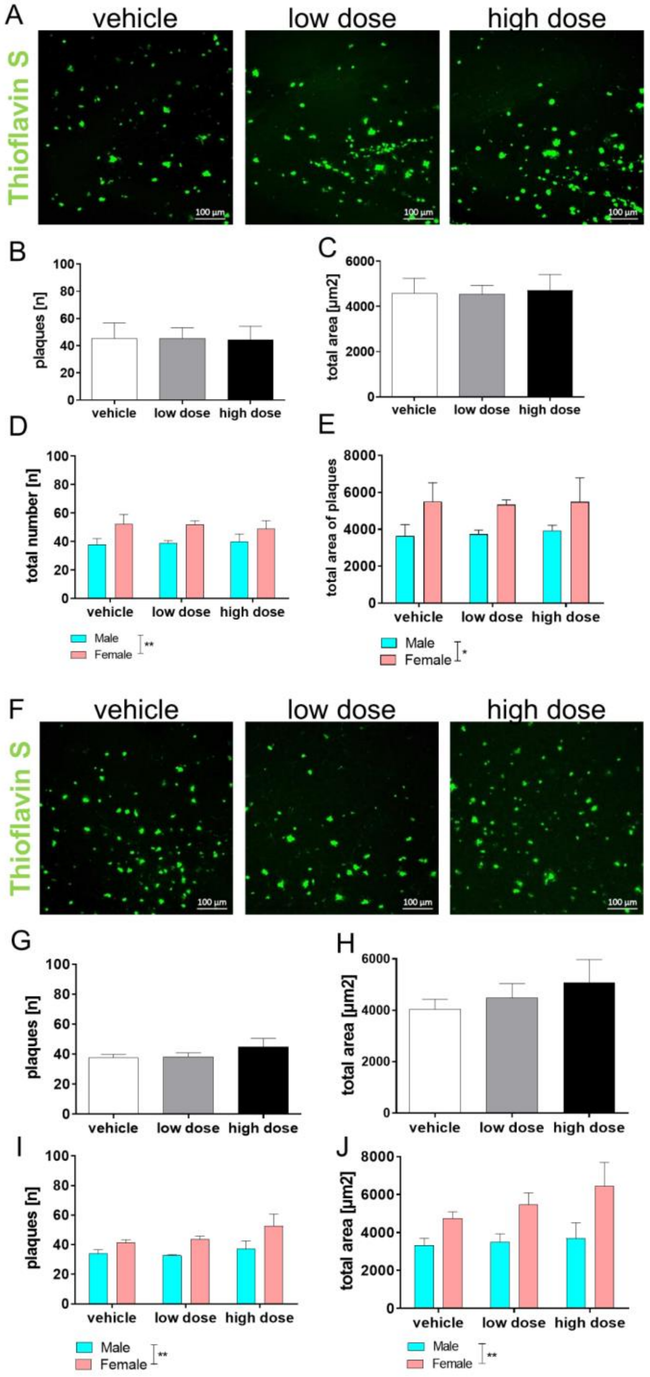

Supplementary figure 4 Plaque analysis in the hippocampus (a-e) and in the cortex (f-j). (a) representative images of hippocampal amyloid beta plaques in animals from all groups. (b) Number of plaques. (c) total area of ThioflavinS staining. (d) total number of plaques separated by sex. (e) total area of ThioflavinS staining in male and female mice. (f) representative images of cortical amyloid beta plaques in animals from all groups. (g) Number of plaques. (h) total area of ThioflavinS staining. (i) total number of plaques separated by sex. (j) total area of ThioflavinS staining in male and female mice in the cortex. Data are shown as mean +/- SEM. One-way ANOVA was performed with Tukey´s post hoc test. P-values < 0.05 were considered significant.

Analysis of MTK effects on CD8+ T-cells in the cortex

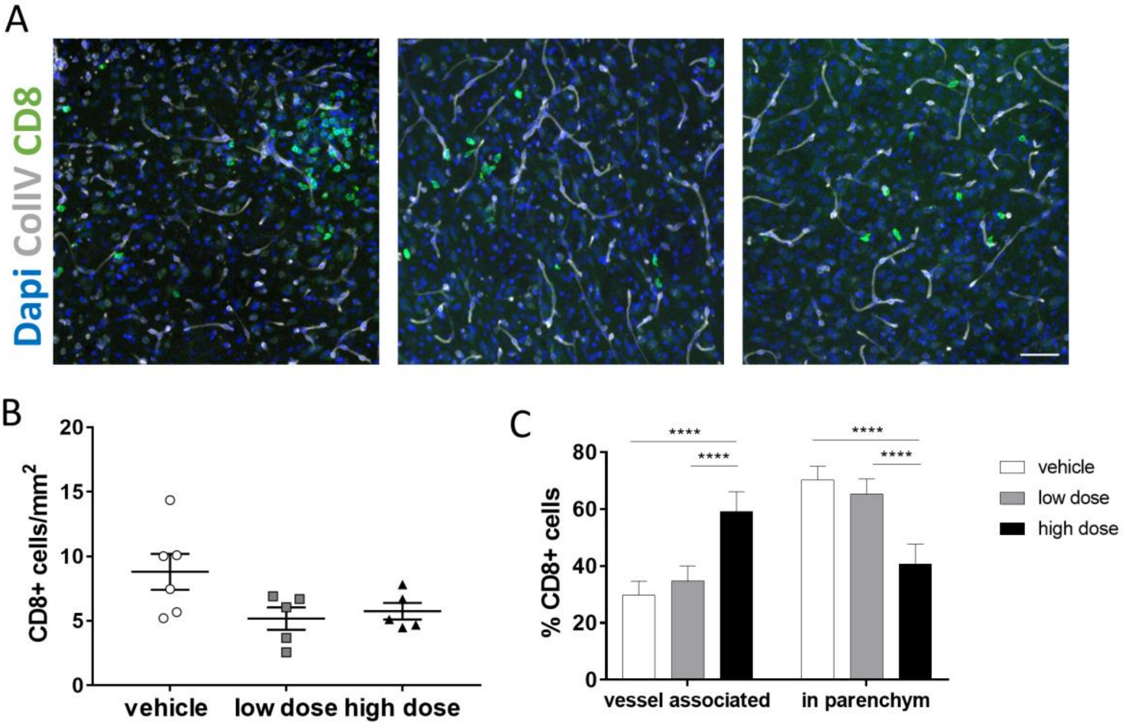

Supplementary figure 5 Immunohistochemical analysis of CD8+ T-cells in the cortex. (a) representative images of parenchymal CD8+ t-cells and ColIV in all groups. (b) number of CD8+ cells per mm2 with and without MTK. (c) Distribution of CD8+ cells between vessel and parenchym among all groups. Data are shown as mean +/- SEM. One-way ANOVA was performed with Tukey´s post hoc test. P-values < 0.05 were considered significant.

MWM data separated by sex

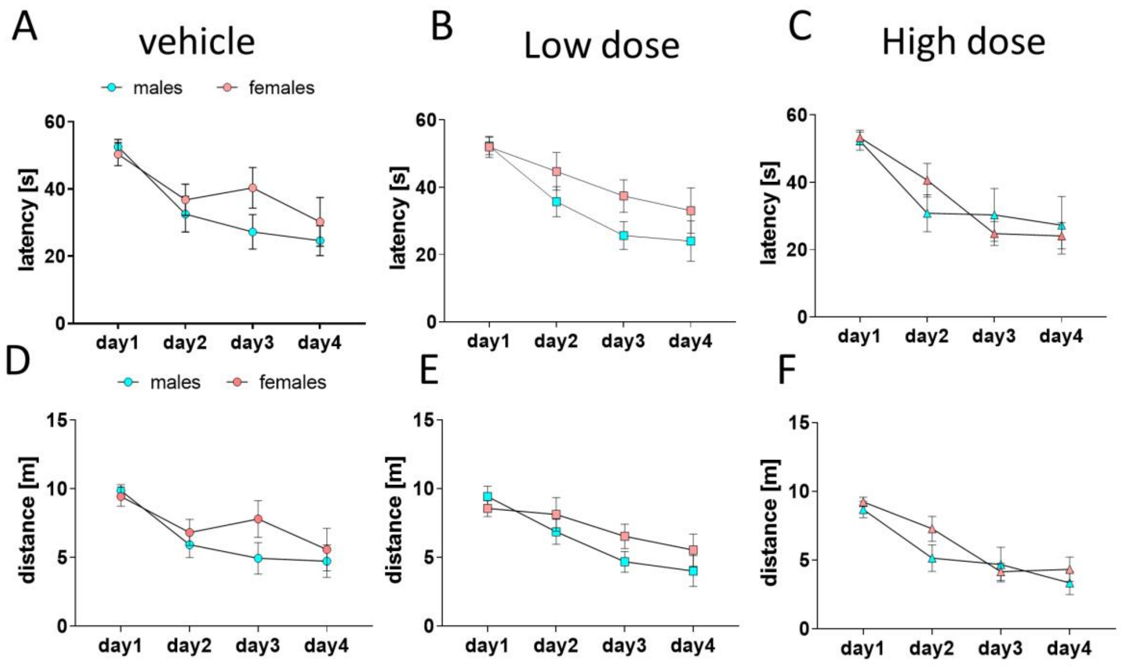

Supplementary figure 6 Behavioral data from MWM analyzed by sex. (a) latency learning curves from male (blue) and female (pink) vehicle treated mice. (b) latency learning curves from low-dose MTK treated mice. (c) latency learning curves from high-dose MTK treated mice. (d) distance curves from male and female vehicle treated mice. (e) distance curves from from low-dose MTK treated mice. (f) distance curves from high-dose MTK treated mice. Data are shown as mean +/- SEM. One-way ANOVA was performed with Tukey´s post hoc test. P-values < 0.05 were considered significant.

Additional behavioral data from Open Field and Elevated Plus Maze

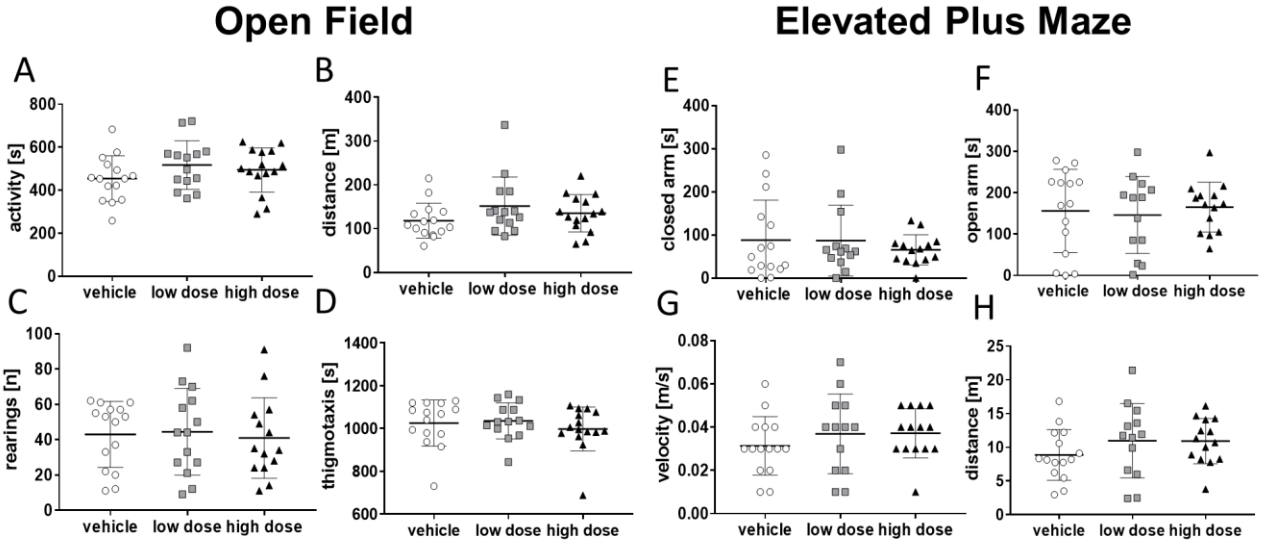

Supplementary figure 7 General behavior and anxiety. (a-d) Mice were tested in the open field for 20 minutes (n=15/group). There was no significant difference between the groups in the parameters of activity (s), distance (m), rearing (n) and thigmotaxis (s). (e-h) Mice were tested in the elevated plus maze (n=14-15/group). There was no significant difference between the groups in the parameters of time spend in closed arm, time spend in open arm, velocity (m/s) and distance (m). Data are shown as mean +/- SEM. Data were tested for normal distribution with the Kolmogorov-Smirnoff test and groups were compared using one-way ANOVA with Tukey´s post hoc test. P-values < 0.05 were considered significant.

Correlation analysis

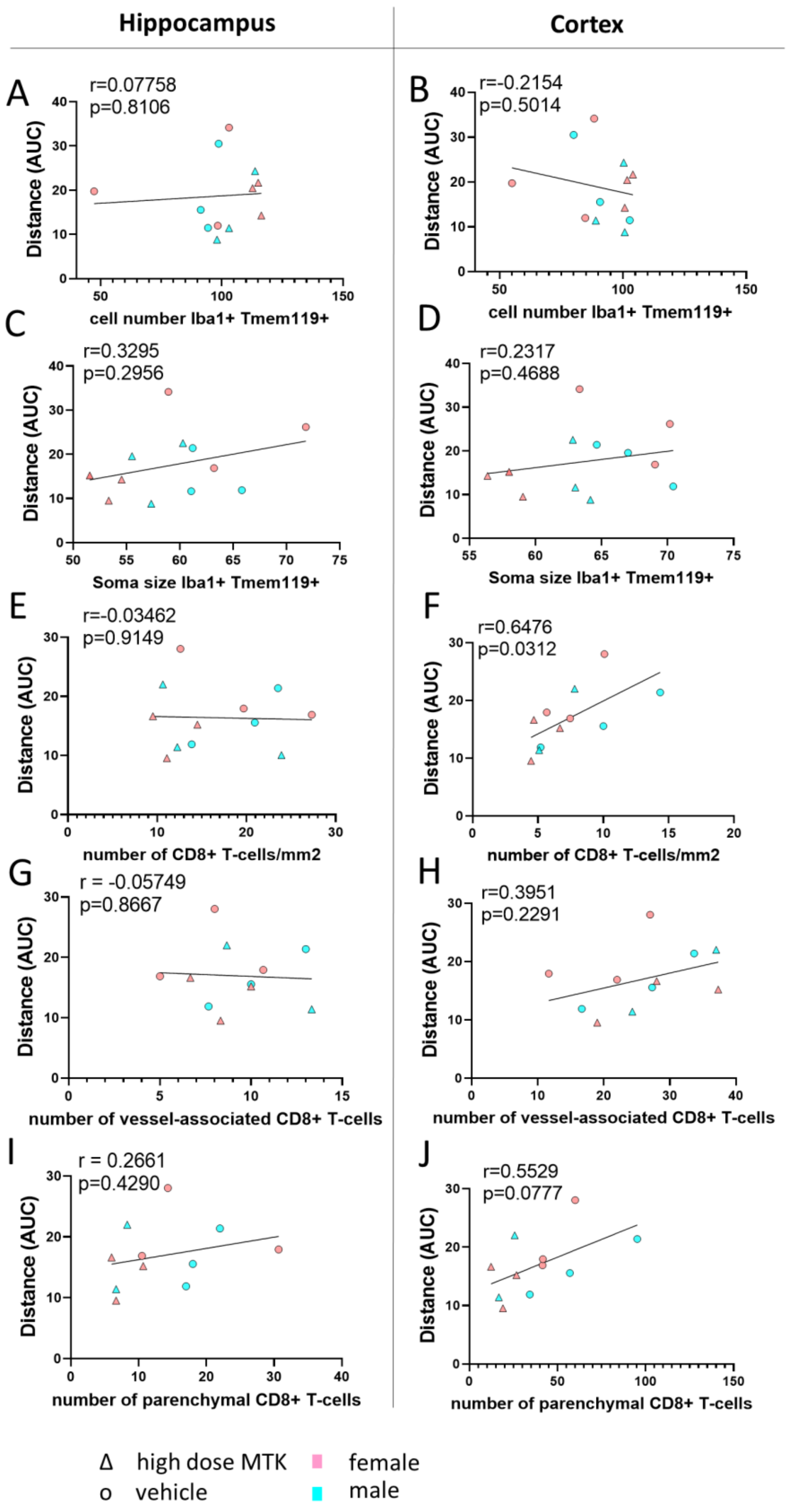

Supplementary figure 8 Pearson´s correlation analysis of cognitive improvement (parameter of distance) with cellular data from hippocampus (a,c,e,g,i) and cortex (b,d,f,h,j).
